# Automatic 360° Mono-Stereo Panorama Generation Using a Cost-Effective Multi-Camera System

**DOI:** 10.3390/s20113097

**Published:** 2020-05-30

**Authors:** Hayat Ullah, Osama Zia, Jun Ho Kim, Kyungjin Han, Jong Weon Lee

**Affiliations:** 1 Mixed Reality and Interaction Lab, Department of Software, Sejong University, Seoul 143-747, Korea; khanh9474@gmail.com (H.U.); osama197@gmail.com (O.Z.); kjinn.han@gmail.com (K.H.); 2 Department of Electrical Information Control, Dong Seoul University, Seongnam 461-140, Korea; heglerkim@du.ac.kr

**Keywords:** fisheye cameras, image sensors, immersive technology, image processing, 360° videos

## Abstract

In recent years, 360° videos have gained the attention of researchers due to their versatility and applications in real-world problems. Also, easy access to different visual sensor kits and easily deployable image acquisition devices have played a vital role in the growth of interest in this area by the research community. Recently, several 360° panorama generation systems have demonstrated reasonable quality generated panoramas. However, these systems are equipped with expensive image sensor networks where multiple cameras are mounted in a circular rig with specific overlapping gaps. In this paper, we propose an economical 360° panorama generation system that generates both mono and stereo panoramas. For mono panorama generation, we present a drone-mounted image acquisition sensor kit that consists of six cameras placed in a circular fashion with optimal overlapping gap. The hardware of our proposed image acquisition system is configured in such way that no user input is required to stitch multiple images. For stereo panorama generation, we propose a lightweight, cost-effective visual sensor kit that uses only three cameras to cover 360° of the surroundings. We also developed stitching software that generates both mono and stereo panoramas using a single image stitching pipeline where the panorama generated by our proposed system is automatically straightened without visible seams. Furthermore, we compared our proposed system with existing mono and stereo contents generation systems in both qualitative and quantitative perspectives, and the comparative measurements obtained verified the effectiveness of our system compared to existing mono and stereo generation systems.

## 1. Introduction

With the rising popularity of virtual reality, 360° panorama generation has become a hot research area. Giant video and search engine servers have started to support 360° videos, thereby attracting many researchers. around the globe. These researchers are contributing to different aspects of 360° videos such as quality enhancement, resolution, and different image acquisition kits to capture 360° videos. The generation of 360° videos requires knowledge of different fields such as image processing, computer graphics, computer vision, virtual reality, and smart city surveillance [1]. Panoramic images have a promising future in virtual tourism [2], parking assistance [3], medical image analysis [4] and digital cities [5]. Moreover, it is a suitable technique to cover wide surveillance areas such as airports, big utility stores, and banks, etc., using 360° video surveillance systems [6]. In order to create panoramic images, there are three different techniques. The first technique for panorama generation uses a single camera that projects the scene from the surroundings through a reflection in a mirror. However, the panorama generated using this approach usually has low resolution. The second technique generates panoramas from the images captured by multiple cameras placed in a circular rig [7]. To use this technique for panorama generation, the positioning of cameras must be set carefully with sufficient overlapping regions between adjacent cameras. The images are then stitched together using feature-based stitching algorithms [8,9]. The third technique creates panoramas using an embedded panoramic generation system [10,11] with resource-constrained devices such as mobile cameras or low-power, hand-held visual sensors. Such techniques first estimate camera motion by continuous tracking of the camera while capturing images from the surroundings and stitch the images using the projected plane of the previously taken image. Although these embedded visual sensor-based approaches are more robust, efficient, and cost-effective for panorama generation, the quality of the panoramas generated using these embedded approaches usually suffer from stitching artifacts such as geometric error (structural error) and photometric error (color distortion).

A massive amount of work has been done in the area of mono panorama generation [12] where the images, captured from different angles of view with different image acquisition kits, are stitched to create a wider field of view image. Nowadays, most of the 360° panorama contents available on the Internet are mono panorama contents. A mono panorama has the same view for both left and right eye. It cannot provide depth information to the user. Most of the existing methods for generating mono panoramas require a lot of user input to achieve better quality results. Such a system is time consuming and difficult for amateur photographers to generate 360° panoramic images. On the other hand, a stereo image consists of two images (left and right) representing a scene from two different points of view that are horizontally displaced. These two images are captured using a twin lens camera system. When the same scene is captured from two different points of view, it gives an illusion of depth to the user. As a result, the output of both left and right image has different representations of image contents in which some content appears closer than others. Similarly, the human visual system is binocular in nature, and the human brain receives different spatial information from both eyes. The FOVs (Field of Views) of both eyes overlap with each other at the center of the eyes, which are then synthesized by the brain to create a single coordinate image. To generate stereo a panorama, expensive equipment [13,14] high computational power, and long processing time are required because the panorama needs to be generated separately for both eyes.

In this paper, we focus on both mono and stereo panorama generation. An efficient and economical approach has been suggested to generate a full 360° panorama (mono and stereo). Our proposed method for mono panorama generation requires no user input to create a panorama. The system is optimized according to the geometry of the camera rig used to gather data. To generate stereo panoramas, we present an effective and reasonable image acquisition setup that uses only three cameras to capture videos from the surroundings. Two cameras cover the front view and one camera is used for capturing the rear view. More specifically, the main contributions of our method are summarized as follows:

- An efficient and cost-effective multi-camera system is proposed for generating 360° panoramas. The precise placement of cameras with enough overlapping gaps for image acquisition makes the panorama generation module fully automatic, which directly stitches images captured with the proposed image acquisition technology without any user interaction. Furthermore, the panorama generated by our system has no visible seams and is automatically straightened.
- Compared to other existing panorama generation systems, the proposed system reduces the computation cost and time complexity by using a portable image acquisition system that uses only six cameras for mono contents generation and three for stereo contents generation.
- The proposed system dominates existing mono and stereo contents generation systems from both qualitative and quantitative perspectives.

The rest of the paper is organized as follows: Section 2, describes the literature of panorama contents generation. The proposed method for both mono and stereo panorama generation is explained in Section 3. Experimental results and the evolution of our approach are discussed in Section 4. Section 5 concludes the paper with some possible future directions.

## 2. Related Work

Creating a new reality (wide field of view) from existing reality (normal field of view) by extending views of a scene by leveraging 360° space allows users to explore content by looking in any direction. The possibilities for viewers to see everything in a scene at a glance has led to the popularity of immersive media in industry, adding new dimensions to human interactions [15] and enhancing newly generated experiences [16]. The researchers are inspired from new technology in HCI (Human Computer Interaction) to use new method for features extraction [17,18], to enhance HCI oriented user experience. The use of immersive technology is also a milestone in the fields of physical science [19] and healthcare [20]. Besides these applications, armed forces are also taking advantage of immersive technology for training [21]. Due to its versatile nature, it has great potential and can bring major changes and revolutions to other industries as well. Panorama generation and image stitching has vast research literature. Over the last decade, the research community has presented many approaches for the generation of wider immersive (FOV) video. The applications of immersive technology are not restricted to virtual reality. Recently, we observed a significant increase in 360° video-driven surveillance systems [22] that provide wider field of view monitoring, thereby improving the performance of overall surveillance. These panoramic videos are usually generated from multiple images captured by special types of cameras [23,24], which are then stitched together to create a single wide field of view frame. These cameras are mounted in a fashion that covers the complete field of view both horizontally (360°) and vertically (180°).

Most of the previous stitching methods were based on feature-based algorithms. For instance, in [25,26] images from multiple cameras were stitched together by extracting features from the images being stitched. These feature-based stitching algorithms have three phases: feature detection, image registration and image blending. In the feature detection phase, key features from the image are detected. During the image registration phase, the images are aligned with each other based on matched features. Different techniques for feature matching have been used in previous work, and among the most well-known ones are Fast Library for Approximate Nearest Neighbors (FLANN), Brute-Force Matcher, and Random Sample Consensus RANSAC [27]. For example, Shi et al. [28] described an image stitching algorithm based on parallax improved feature blocks (PIFB). First, each image is divided into multiple feature blocks using a fuzzy c-means algorithm and a characteristic descriptor of each feature block is extracted using scale-invariant feature transform (SIFT). Second, the feature mapping and homography are calculated using the feature points in the feature block. Finally, a ghosting free image is achieved by optimizing overlapping regions. However, their proposed approach only focused on a ghosting error, and cannot eliminate other stitching errors such as blending error and structure inconsistency error. Chi et al. [29] proposed a line-point feature-based stitching algorithm. Their proposed system used a complex stitching strategy, where they first refine the alignment of lower-texture regions and then performed super pixel segmentation to enhance the unreliable point correspondences. To remove the visible seams from the resultant panorama, an image blending operation is applied to the output panoramic image [30,31,32]. The visual quality of the output panorama depends on the overlapping regions. We can achieve a high-quality panorama without any stitching errors with sufficient overlapping regions. Shimizu et al. [33] presented a video stitching method approach based on motion tracking. To track the global motion for each input video, first, the projection matrix is calculated between stitched frames and then fine adjustment is performed to obtain the desired resultant stitched images. However, their proposed system is limited to stitched only two frames simultaneously and cannot be used for creating high-level 360° contents from more than two images.

Besides the aforementioned techniques, several embedded approaches have been proposed for panorama generation tasks. Kim et al. [34] proposed an image stitching algorithm for mobile-oriented multimedia devices. The stitched images were obtained through an optimal seam near the transition region between images. They adopted a color blending algorithm for the removal of visible seams at the boundaries of the overlapping regions of stitched images. However, the panorama generated by their proposed system have color inconsistency error near stitching regions that condense the perceptual quality of panoramic contents. A similar approach is presented by Kim et al. [35], where the authors first find the optimal seam between adjacent frames and apply content-aware adaptive blending to stitched frames, which greatly reduced color discontinuity and obtained a good quality stitched video. Their proposed system finds the optimal seam based on moving objects, hence a minor error during motion estimation can affect the performance of their system. To acquire accurate camera coordination, Guan et al. [36] proposed a polar coordinate transformation approach for imaging navigation sensors that utilized the polarization information of polarized angle images. First, they set a polar coordinate system on the image of angle of polarization. Next, they estimated the corresponding point of the single pixel value and the rotational angle of solar meridian based on a trigonometric relationship. To evaluate the camera calibration errors, Chen et al. [37] used a multi-camera vision system to retrieve the visual information of static and dynamic objects. They applied both local and global calibration to obtain multi-camera correlation and performed image stitching operations to acquire filtered global points. Furthermore, they used a point correction algorithm to optimize the parameters and improve the stitching results. Similarly, Tang et al. [38] developed a real-time detection framework for surface deformation and strain in recycled concrete-filled steel. They used dynamic surface tracking, automatic calibration, and mathematical models to combine the four-ocular visual coordinates and a point cloud. Finally, they recreated 3D deformation surfaces using multi-ocular vision coordinates, point cloud registration, and image preprocessing. Lin et al. [39] proposed an algorithm based on RGB depth for citrus detection and localization in orchard environments. First, they segment the background using depth filters and Bayes-classifier and then a density clustering method is used to cluster the adjacent points in the filtered RGB-D images. Finally, a multi-domain (gradient, color, and geometry features) feature-based support vector machine is used to classify pixel values for final segmentation. Tang et al. [40] also presented a detailed discussion about the recent progress of machine-vision technology and current main challenges. They discussed state-of-the-art vision-based approaches to civil infrastructure condition assessment and mentioned the key limitations to these methods. Joshi et al. [41] proposed adaptive selection to minimize the alignment error in stitched images (panoramic images). They also smooth the final panorama using 2D video stabilization. Although this method can be used for mobile devices in real time, it cannot be applied directly to 360° videos. Osama et al. [7] proposed a hardware-based approach to automatic panorama generation, where images are captured by fisheye cameras mounted on a drone, and the captured images are stitched together using a feature-based stitching algorithm. The authors claimed that their proposed method generates high-quality panoramas without any post processing. In the immersive media industry, most panoramic images are created using pre-built software for image stitching such as Autostitch [42], Panoweaver [43], and Kolor Autopano [44]. These panoramic content generation software applications are difficult to use and require enough experience. Since, the previous panoramic contents generation systems either focused on hardware (such as number of required cameras) or software part (such as quality of generated panoramic contents). Different from these methods, in this paper we present an automatic mano-stereo contents generation framework. The proposed system not only generates high quality panorama but also reduce the time complexity and number of required cameras for creating 360° contents.

For mono panorama generation, we proposed a unique hardware design that make the stitching process automatic with less user input. The stitching process of our system is fully automatic, which makes the overall system a robust and real time system. For stereo panorama generation we presented an innovative system that uses a static camera rig that contains three fisheye cameras. Since the system requires only three cameras, it is much more economical than existing stereo panorama generation systems.

## 3. Proposed Methodology 

**Figure 1:**
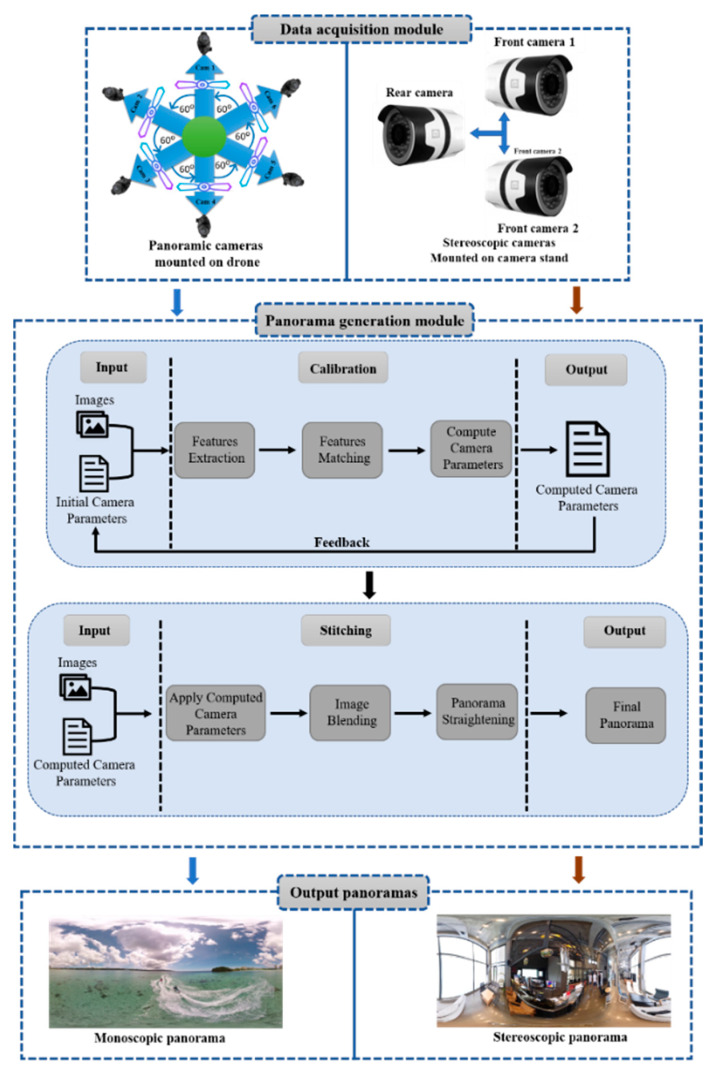
A detailed overview of the proposed panorama generation framework. The proposed framework involves two main steps including data acquisition and panorama generation modules. The ***data acquisition module*** uses two different image acquisition systems (five cameras for mono data acquisition and three for stereo data acquisition) to acquire images for mono and stereo content generation. The ***panorama generation module*** first performs a camera calibration process to optimize the camera parameters, and then stitches multiple input images into a single panoramic image using feature extraction, feature matching, and image blending.

In this paper, we present a dual-feature panorama generation system that generates both mono and stereo panoramas. The proposed method mainly consists of two phases: firstly, using the proposed camera model, the data for both mono and stereo is generated and forward to the panorama generation module. Secondly, for image stitching, cameras parameters are computed using initial guesses, as shown in Figure 1, which shows the complete workflow of our proposed method. Each component of the proposed framework is described in a separate section with detailed explanation. The parameters used by the proposed method for input and output operations are listed in Table 1. 

### 3.1. Data Acquisition

The hardware setup contains two camera models, one for mono data generation and the other for stereo data generation. Both camera models capture video data which are then passed on to the panorama generation module. The process of data acquisition for both mono and stereo is explained in the next subsections. 

#### 3.1.1. Mono Data Generation

The hardware proposed for mono data contains six cameras that are mounted on a drone. Each camera is attached to the drone’s leg, and there is a 30° overlapping gap between two adjacent cameras. Besides 30° overlapping, each single camera covers a 60° view of the external surroundings. The images taken with these six cameras are then passed on to the panorama generation module. The proposed system is automatic and no user input is required, and the resultant panorama does not require any post processing. Thus, in the panorama generation phase there is no need for post processing to remove unwanted artifacts (images of the drone itself). For every panorama generation module, an efficient overlapping region between the images captured by the adjacent cameras is very important, which we achieve with the FOVs (60°) between each adjacent camera in circular the rig. For the adjustment of cameras position, we used the Y-up coordinate system that transforms the points of the camera coordinates into a real-world coordinate system. Generally, the Y-up coordinate system has three coordinates, namely *x*-axis, *y*-axis, and *z*-axis, where x, y, and z represent width, height, and depth in the real world. Initially, the values for these coordinates are set to (0,0,0), and are later updated by translating the position of the cameras. The position of a camera’s is translated based on the camera viewpoint towards the scene to be captured. 

**Figure 2:**
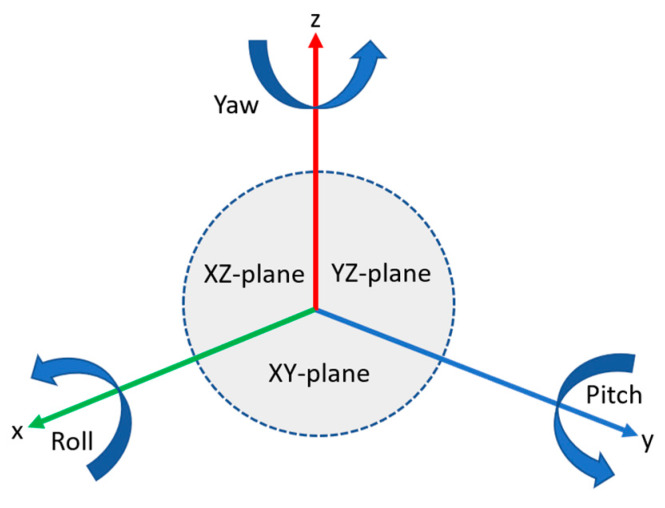
Diagram of the Y-up coordinate system.

**Figure 3:**
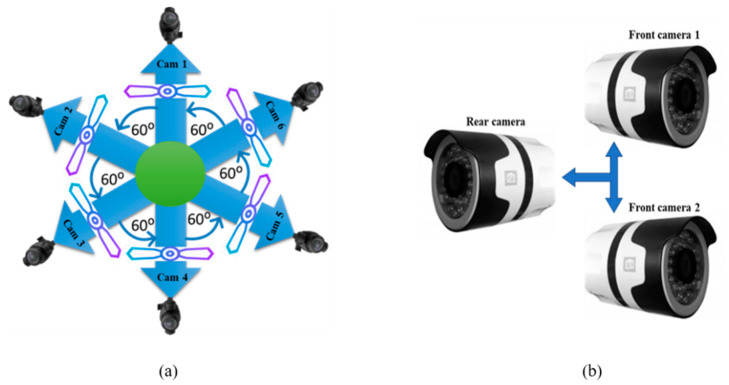
The proposed camera setup for panorama generation: (**a**) camera step for mono panorama generation, (**b**) camera setup for stereo panorama generation.

Figure 2 shows the Y-up coordinate system, where roll is rotation around the *x*-axis, pitch is rotation around the *y*-axis, and yaw is rotation around the *z*-axis. In the initial orientation of mono camera parameters, cameras are rotated only around the *z*-axis where x and y coordinates are remine same with initial values, which affects only the yaw values of the Y-up coordinate system, as listed in Table 2. In Table 2, positive yaw values for camera 1–4 represent the clockwise rotation of cameras around the *z*-axis, whereas the negative yaw values for camera 5 and 6 represent anticlockwise rotation around the *z*-axis. The camera configuration and placement for mono and stereo data acquisition is depicted in Figure 3a,b, respectively. 

#### 3.1.2. Stereo Data Generation

A panoramic view is created from stereo data where one panorama is generated for the left eye and another panorama is generated for the right eye. Numerous hardware-based approaches have been proposed. Most of these approaches are expensive due to the use of multiple cameras. In this paper, we present cost-effective hardware for generating stereo panoramas. Our proposed method uses only three cameras for acquiring data, two cameras cover the front view and one camera covered the rear view (back view). As the front view is more important than the rear view, we have designed a hardware system that captures the front view as a stereo image and the rear view as a normal 2D image. While generating stereo data, we use a wider FOV lens for the rear camera because the two front cameras are placed very close together. So, images captured by these cameras have some unwanted artifacts. These artifacts are automatically masked by the wider FOV images from the rear camera. The placement of cameras in the camera rig is shown in Figure 3b. All the cameras are fitted with a custom fisheye lens. The FOVs of each lens are given in Table 3.

### 3.2. Panorama Generation Module

This section presents the technical details of the panorama generation module along with its main components, where each component is described in a separate section. Different from existing panoramic contents generation systems, our proposed framework is capable to generate high quality mono and stereo panoramas using a simple image stitching pipeline. For mono panoramas, the images captured by drone with the proposed hardware system are passed through a panorama generation pipeline with multiple steps such as feature extraction, feature matching, image stitching, and image blending. The unique feature of the hardware design is the automatic stitching without any post-processing steps. For stereo panoramas, we have proposed a hardware-based solution that produces a stereo panorama using only three cameras. Out of these three fisheye cameras, two cameras form a stereo pair to cover the front view while the third camera covers the rear view. In a stereo panorama, the front view is more important than the rear view. In this regard, we have designed a camera rig that captures the front view in stereo and the rear view in mono. The entire panorama generation process consists of two sub-modules (camera calibration and image stitching). The output of sub-module 1 is the input for sub-module 2. The main components of these submodules are discussed in detail in a separate section.

#### 3.2.1. Camera Calibration

The main purpose of camera calibration [45] is to map the camera coordinates to the world coordinate system. Generally, this mapping requires the computation of two types of parameters, including intrinsic and extrinsic parameters. The intrinsic parameters are camera lens parameters, whereas extrinsic parameters are the camera orientation parameters. Initially, the camera parameters (both intrinsic and extrinsic) are roughly assigned to each camera, which are then optimized iteratively for individual cameras using reprojection error and residual error. The initial camera parameters help the camera calibration process for fast convergence to a solution. The overall camera calibration phase can be dived into three parts, namely feature extraction, feature matching, and computation of camera parameters. The stepwise mechanism of camera calibration is given in Algorithm 1.

##### Feature Extraction

In the camera calibration module, we first extract consistent features from images that are going to be stitched. For stitching, we use invariant features rather than traditional features (such as HOG and LBP features) because invariant features are more robust in frames with varying orientation [46]. By considering these assumptions, we proposed Oriented FAST and Rotated BRIEF (ORB) as a feature descriptor for feature extraction [47]. ORB is computationally efficient and fast compared to the SIFT descriptor mostly used for panorama generation [48,49].

##### Feature Matching

The second step involves features matching, where features of adjacent images are compared and obtained the best matches. For feature matching we used Random Sample Consensus (RANSAC) technique, RANSAC is a sampling approach to estimating homography H that uses a set of random samples to find the best matches. First it selects a set of consistent features and then computes the homography H between two images using the direct liner transformation (DLT) method [50].

##### Optimization of Camera Parameters

To calculate the optimal camera parameters, we forward the random guess values with input images as an initial camera parameter. Both the intrinsic and extrinsic camera parameters are optimized in an iterative fashion. For parameter optimization, we used the bundle adjustment technique, which determines consistent matches between adjacent images. In order to find the most accurate matches, images with the best matches are selected for processing at each iteration. Mathematically, both the intrinsic and extrinsic parameters can be expressed by [51]:

(1) $$M_{intrinsic}=\left(\begin{array}{ccc} fx & 0 & cx\\ 0 & fy & cy\\ 0 & 0 & 1 \end{array}\right)$$

(2) $$M_{extrinsic}=\left(\begin{array}{ccc} r11 & r12 & r13-R^{T}{}_{1}T\\ r21 & r22 & r23-R^{T}{}_{1}T\\ r31 & r32 & r33-R^{T}{}_{1}T \end{array}\right)$$

In Equation (1), *f_x_ and f_y_* are the focal length of *x* and *y* coordinates, and *cx* and *cy* are the principal focus coordinates. Equation (2), gives the extrinsic parameters, that determine the location in real-world coordinates. The rotation value *R*_3 × 3_ is used to find the optimal orientation of cameras with respect to a real-world frame, where *T*_3 × 1_ is a translation vector that defines the position of cameras in the real-world coordinates. Both intrinsic and extrinsic parameters can be combined as a unified camera computation model using Equation (3):

(3) $$q_{cam}=sM_{intrinsic}*M_{extrinsic}*Q_{cam}$$

In Equation (3), *M_intrinsic_* and *M_extrinsic_* are the intrinsic and extrinsic parameters, and *s* is the scaling factor value. *Q_cam_* represents the corresponding 3D points (*x*,*y*,*z*,1) of each camera in real-world coordinates and *q_cam_* is the 2D point (*m*,*n*,1) of the image surface. For better understanding, Equation (3) can be rewritten as:

(4) $$\left(\begin{array}{c} m\\ n\\ 1 \end{array}\right)=sM_{intrinsic}*M_{extrinsic}*\left(\begin{array}{c} x\\ y\\ z\\ 1 \end{array}\right)$$

During the computation of camera parameters, parameter optimization is iteratively evaluated using mean reprojection error. The reprojection error determines the distance between the estimated projection points $\hat{x}$ and the actual projection points *x*. The reprojection error for parameter optimization can be express by:

(5) $$Error_{reprojection}=\sum_{i}d{\left(x_{i},{\hat{x}}_{i}\right)}^{2}+d{\left(x_{i}{}^{\prime },{\hat{x}}_{i}{}^{\prime }\right)}^{2}$$

**Figure 4:**
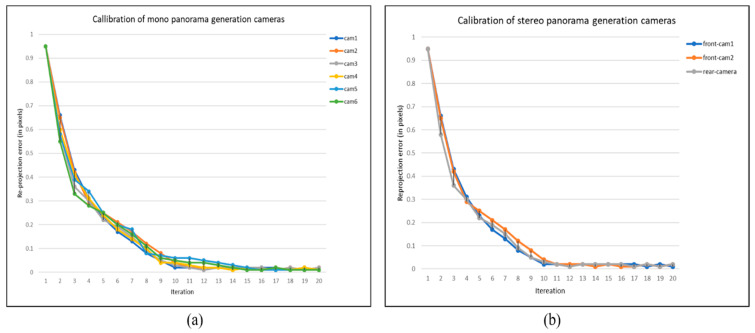
The optimization of camera parameter. (**a**) Reprojection error analysis of mono cameras, (**b**) Reprojection error analysis of stereo cameras.

In Equation (5), *x_i_* and ${\hat{x}}_{i}$ are the actual and estimated projection points, while *x_i_′* and ${\hat{x}}_{i}{}^{\prime }$ are the imperfect and perfect matched points, respectively, and d is the Euclidean distance that calculate the difference between *(x_i_′, ${\hat{x}}_{i}$)* and (*x_i_′*, ${\hat{x}}_{i}{}^{\prime }$). The reprojection error is calculated iteratively *i* times, and the value of *i* is not fixed since it depends on how rapidly the camera parameters are going to converge. The reprojection errors during the camera calibration phase for both mono and stereo content generation cameras are depicted in Figure 4a,b respectively. It can be seen that the parameters for each camera are optimized after each iteration with the feedback of refined parameters from immediately last iteration.

#### 3.2.2. Image Stitching

Image stitching is the process of combining multiple images to make a wider field of view image. Generally, it is divided into two main steps. First, the two images are registered by matching the detected consistent features to determine their overlapping region. Second, the images are wrapped and stitched together based on the optimized camera parameters calculated in the image calibration phase. Finally, an image blending operation is performed to eliminate the visible seams at the boundaries of the stitched regions. The step by step mechanism for image stitching is given in Algorithm 2.

##### Image Alignment

In image stitching pipeline, we first align the adjacent unstitched images based on best matched features. For image alignment, we compute the homography *H* (3 × 3 matrix) between adjacent images that warps one image with respect to another image. For instance, point *Pʹ (xʹ, yʹ,1)* of image 1 and point *P (x, y,1)* of image 2 can be corelate using homography Equation (6). To calculate a correct homography between two images, there must be at least 4 best matches (four coordinates) between the images to be aligned:

(6) $$P=H*P^{\prime }$$

where *H* is a 3 × 3 matrix as given in Equation (7):

(7) $$H=\left(\begin{array}{ccc} h_{11} & h_{12} & h_{13}\\ h_{21} & h_{22} & h_{23}\\ h_{31} & h_{32} & h_{33} \end{array}\right)$$

The homography computation process determines the refine coordinates and replace the old coordinate system of the image with new coordinate system. Finally, the processed images are warp to each other based on computed homography.

##### Image Blending

The final phase of panorama generation is image blending, which remove the visible seams at the boundaries of adjacent images. To remove these visible seams, variety of image blending techniques have been proposed including Average Blending [31], Alpha Blending [52], Pyramid Blending [53], Poisson Blending [54], and multi-band blending [55,56]. Inspired from the efficiency of multi-band blending technique for image mosaicking in [56], we used multi-band blending technique for image blending. First, it generates a Laplacian pyramid and then estimate the Region of multi-band blending technique for image mosaicking in [56], we used multi-band blending technique for image blending. First, it generates a Laplacian pyramid and then estimate the Region of Interest (ROI) to be blended and project the image on its adjacent image using estimated ROI with best matches. To obtain the final results, all the blended images from different levels are linearly combined as a single image. Since, there are different levels where each level can be considered a mapping function between the stitched images and levels of pyramid. Mathematically, multi-band blending can be written as:

(8) $$\beta =\sum_{i=1}^{l}exp(\Delta_{i})$$

Here, the number of layer is denoted by *l*, exp is a function which restore the image to its original resolution. Where *Δ_i_* is defined as:
**Algorithm 1** Camera Calibration Steps**Input:** 1) Images Im || Is    2) Initial camera parameters ICP    *note: Im and Is are the images taken with the proposed mono and stereo cameras. Where || demonstrates that input will either be Im or Is**Output:** Computed camera parameters CCP**Steps:****while (Im || Is)** 1: Extract consistent features, £c ← ORB (Im_i_, Im_i+1_, Im_i+2_, Im_i+3_, Im_i+4_, Im_i+5_) 2: Feature matching, Im_f_ ← RANSAC (£_c_) 3: Homography calculation, Fm_f_ ← H(Im_f_) 4: Computing camera parameters, CCP ← Φ (Fm_f_) **end while**


**Algorithm 2** Image Stitching Steps**Input:** 1: Images Im || Is    2: Computed camera parameters CCP **Output:** Panoramic image թ
**Steps:**
**while (Im || Is)** 1: Image wrapping, w_i_ ← Щ (Im_i_, Im_i+1_, Im_i+2_, Im_i+3_, Im_i+4_, Im_i+5,_ CCP) 2: Image blending, I_blend_ ← βmulti-band (w_i,_ w_i+1_, w_i+2_, w_i+3_, w_i+4_, w_i+5_) 3: Panorama straightening, թ ← ζ_p_ (I_blend(i)_, I_blend(i+1)_, I_blend(i+2)_, I_blend(i+3)_, I_blend(i+4)_, I_blend(i+5)_) **end while**


(9) $$\Delta_{i}=\sum_{j=1}^{n}\Omega_{j}^{i}\Theta_{j}^{i}$$

Here, *Ω_j_^i^* is the *j^th^* Gaussian pyramid at level *i*, similarly *Θ_j_^i^* is the *j^th^* Laplacian pyramid et level *l.*

##### Panorama Straightening

As feature matching and computation of camera parameters in camera calibration phase helps the image stitching process during panorama generation. However, the resultant panoramas usually have wavy artifacts that significantly reduce the perceptual quality. These wavy artifacts are occurred due to misalignment of adjacent cameras, to remove these wavy affects, we used global rotation technique [50] for panorama straightening and obtained a high-quality straight panorama.

## 4. Experimental Results

In this section, we present details about the experimental assessment of both mono and stereo panorama generation. The proposed method is implemented in C++ using Nvidia Stitching SDK on a machine equipped with a GeForce-Titan-X 1060 GPU (6 GB), 3.3 GHz processor, and 8 GB main memory (Random Access Memory, or RAM for short). Furthermore, we compared the proposed system with existing mono and stereo panorama generation systems. 

### 4.1. Mono Panorama Results

In this section we assess the results of the mono panorama. The images captured from the six fisheye cameras are first passed through a data preparation module. After performing some preprocessing operations, these captured images are then fed into the panorama generation module. These cameras are mounted on the legs of a drone, where each camera is attached with drone leg. The placement of each camera is done in such way that they have a sufficient overlapping region, which helps the image stitching process during the panorama generation phase.

**Figure 5:**
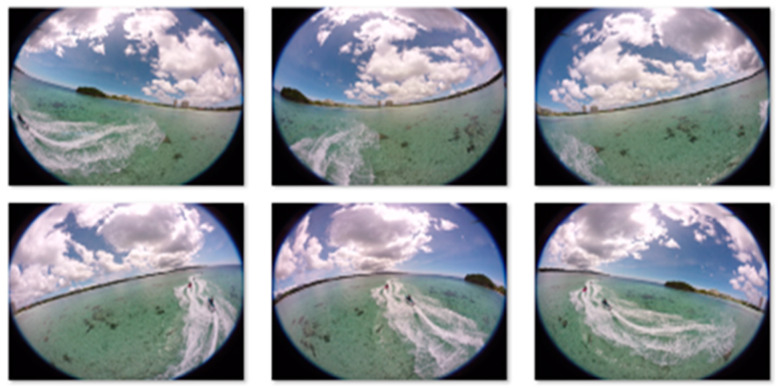
Representative captured images from drone-mounted cameras for mono panorama generation.

**Figure 6:**
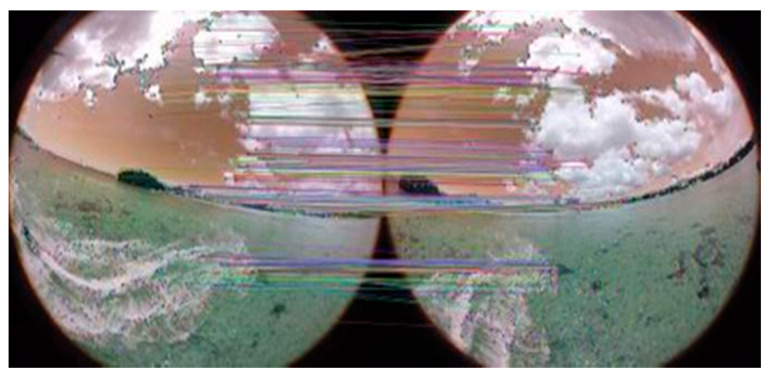
Feature matching between adjacent images.

The initial camera parameters are guessed using the initial orientation of cameras in the rig. The initial camera parameters assist the system while computing the refined camera parameters. These camera parameters are then used to improve the calibration of cameras, which boosts the overall performance of the system. Images captured using the proposed cameras are shown in Figure 5. These images captured by the proposed camera system ensure that the drone is not part of any camera view. It enables the proposed panorama generation framework to create an automatic panorama without any post processing. The captured mono images are then stitched together and create a panorama based on consistent matched features. The feature matching process is shown in Figure 6.

Once the feature mapping process between adjacent images is completed, these images are then stitched together and passed through an image blending phase that removes the visible seams from the resultant panorama using the multi-band blending method [57]. Multi-band blending first computes the ROI of each input image, and then projects the input images according to the corresponding ROIs. After image projection, the next step computes the blending masks and generates a gaussian pyramid for each mask to blend the ROIs. Finally, the resultant panorama is forward to panorama straightening module, which remove the wavy artifacts from the input panorama and obtained artifact-free straight panorama using global rotation technique [58].

#### Comparison with State-of-the-Art Mono Panorama Generation Systems

**Figure 7:**
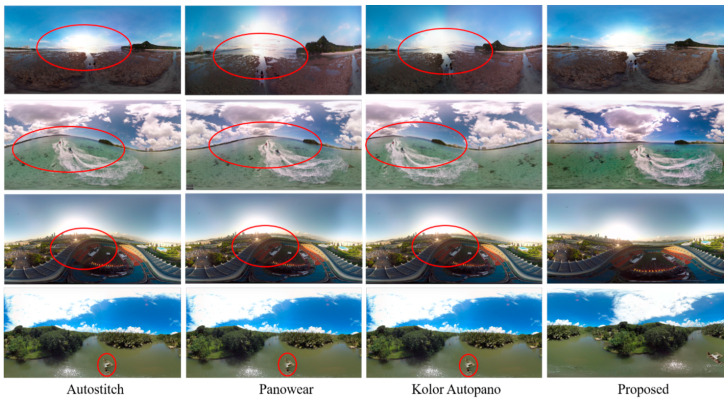
Visual comparison of panoramas generated by our proposed system with existing manual panorama generation systems.

**Figure 8:**
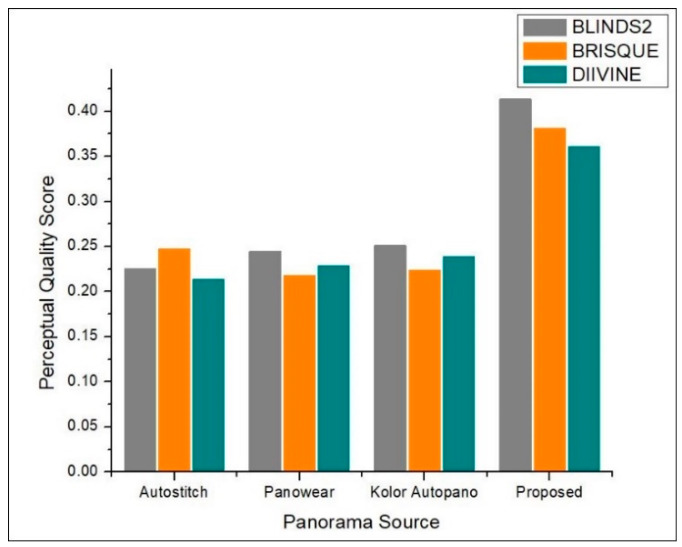
Quantitative performance evaluation of our proposed system compared to existing manual panorama generation software programs.

This section details the experimental evaluation of the proposed system from three perspectives including qualitative, quantitative, and efficiency of hardware. First, the results obtained by our proposed system are visually compared with state-of-the-art stitching software including Autostitch [42], Panowear [43], and Kolor Autopano [44]. The visual comparison is shown in Figure 7, where it can be seen in the top three rows that panoramas generated by [42,43,44] have wavy artifacts highlight by red circles, while the panorama generated by our proposed system has no wavy artifacts and looks better than the rest of the panoramas generated by stitching software. Similarly, in the bottom row, the three left-most panoramas have parallax artifacts highlighted by red circles, whereas the panorama generated by our system has no parallax artifacts. Also, we compared the quantitative results obtained by our system with state-of-the-art systems [42,43,44]. For quantitative evaluation, we compared the proposed system with [42,43,44] in terms of quality score. Since we are dealing with panoramic images where it is sometimes impossible to have a reference panoramic image in advance, we selected three no-reference Image Quality Assessment (IQA) metrics including BLINDS2 [59], BRISQUE [60], and DIIVINE [61]. We then computed the quality score of panoramic images generated by our proposed system along with other three image stitching software programs [42,43,44] using the aforementioned metrics. Figure 8 shows an objective evaluation of our proposed system compared to state-of-the-art image stitching software programs. It can be seen that our proposed system dominated the existing manual panorama generation systems regarding the perceptual quality of the created panorama. Finally, we compared the proposed system with existing systems [62,63] in terms of number of cameras, panorama resolution, stitching artifacts, and stitching time. A comparative analysis of our proposed system with other mono contents generation systems is presented in Table 4. The comparative measures in Table 4 verify that our proposed system generates artifact-free panoramas with an average running time 0.031, which is the least time taken by any comparative method. Whereas the panoramas generated by other comparative methods have stitching artifacts, and these systems also have greater time complexity.

### 4.2. Stereo Panorama Results

**Figure 9:**
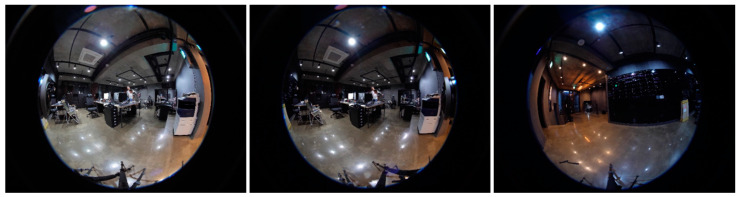
Image from the left-front camera (**left**), image from right-front camera (**center**), image from rear camera (**right**).

**Figure 10:**
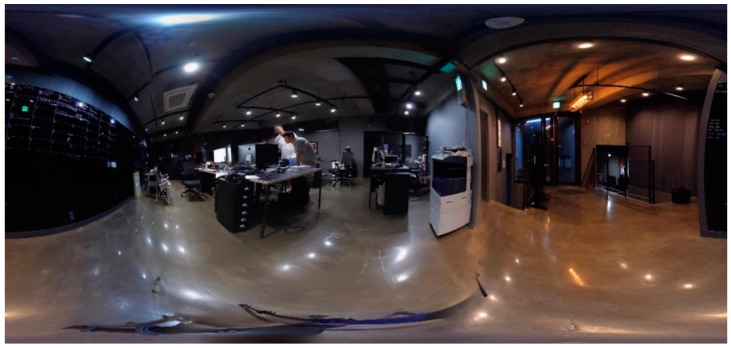
The Left-view stereo panorama created by our proposed system.

**Figure 11:**
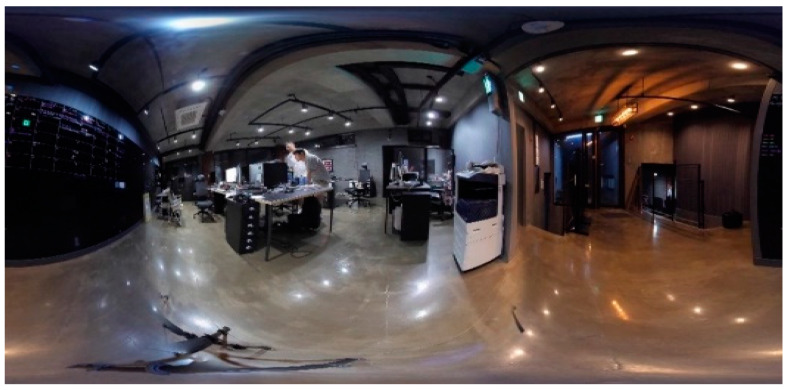
The Right-view stereo panorama created by our proposed system.

**Figure 12:**
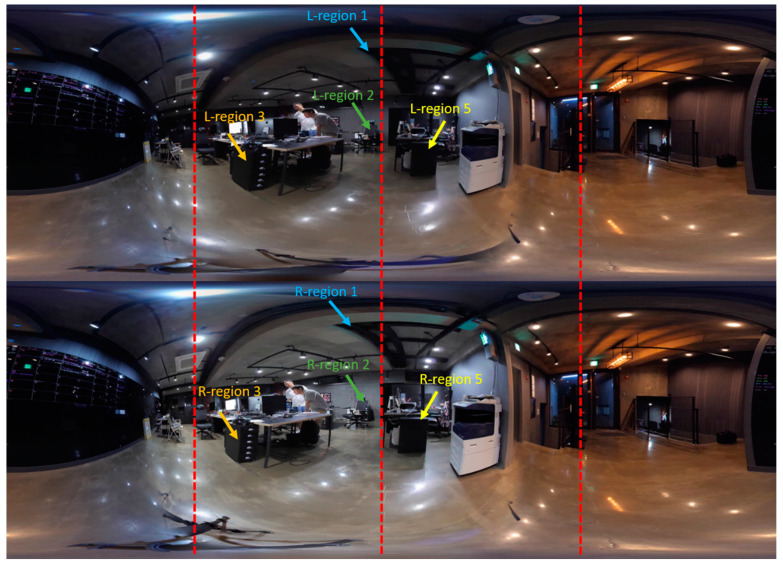
The final 3D stereo panorama generated by our proposed system, which provides a 3D view by stacking the left stereo panorama on the top of the right stereo panorama. Since the stereo panorama has different views for the left and right eye, the perceptual differences for both eyes are demonstrated in the left and right stereo panorama using certain regions. Where the perceptual difference for each selected region in both the left and right stereo panorama is highlighted using arrows, the same regions in different panoramas (left and right) are highlighted with the same color.

In this section, we evaluate the results of the stereo panorama. The proposed camera system for stereo panorama generation is different from the mono camera system, where we proposed a hardware design that contains three cameras, two cameras for capturing the front view while one camera is used for capturing the rear (back) view. The FOV of the rear camera lens is different from that of the front cameras. The reason for using a wider FOV lens for the rear camera is that the front two cameras are placed close to each other, and as a result images captured by these cameras have some unwanted artifacts. These artifacts are automatically masked by the wider FOV image from the rear camera. The images captured by these three cameras are shown in Figure 9. In order to create a stereo panorama, we need to stitch two panoramas, a left panorama and a right panorama. To create the left panorama, the image captured by the left-front camera is stitched with the image from rear camera. Similarly, the right panorama is created by stitching the image captured by the right-front camera with the image from the rear camera. The resultant left panorama is shown in Figure 10 and the right panorama is shown in Figure 11. After stitching the left and right panoramas, the final step is to stack the left and right panoramas vertically in a top-down configuration to form a stereo panorama. The left panorama is placed on top while the right panorama is at the bottom, as shown in Figure 12. The central dotted red lines in Figure 12 show that objects don’t line up in the central region. In order to highlight the perceptual difference between left and right panoramas near the central red dotted line, we select five regions from both the left and right panorama to spot the difference near the line. Among the five selected regions, four regions are on left and one is on the right of the central dotted line. Each specific region has a different view in the left and right panoramas. For example, the object size in region 3 of left panorama L-region3 is different as compare to right panorama R-region3. Similarly, the position of the chair in region 2 of left panorama L-region2 is different from right panorama R-region2. These perceptual differences in viewpoints give the illusion of depth when these panoramic images are viewed through a Head Mounted Display (HMD) device. the left and right dotted lines show that the view captured by the rear camera is same for both the left and right panorama. 

#### Comparison with State-of-the-Art Stereo Panorama Generation Systems

**Figure 13:**
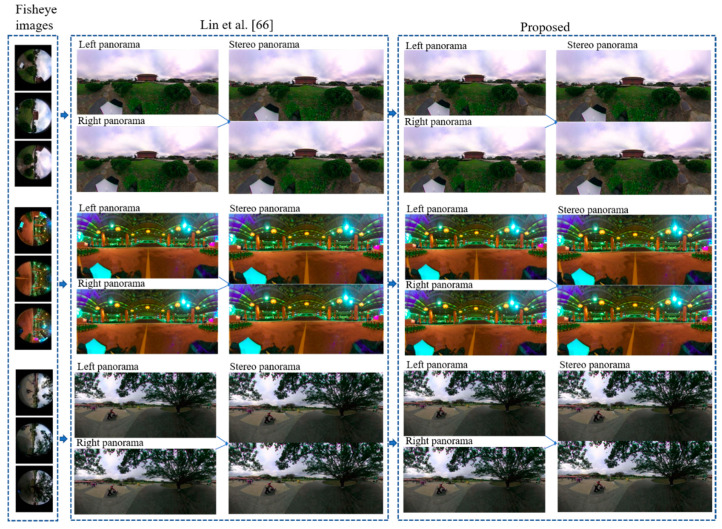
The visual comparison of stereo contents generated by Lin et al. [66] and our proposed system.

This section presents the detailed empirical analysis of our proposed system with existing stereo panorama generation systems in terms of both qualitative and quantitative perspectives. For qualitative evaluation, the visual comparison has been conducted where we compared the stereo panoramas generated by our proposed with stereo panoramas generated by system proposed in [66]. Their proposed system used four cameras to generate stereo panorama, while we used three cameras to create 360° stereo contents. The visual comparison of our proposed system with stereo contents creation system [66] is shown in Figure 13, where it can be seem that our proposed system generates high quality stereo panorama using only three cameras. Further, we evaluated the quantitative performance of our proposed system, where we estimate the perceptual quality of stereo panorama using three image fidelity metrices including Peak Signal-to-Nosie Ration (PSNR), Structural Similarity Index (SSIM), and Root Mean Square Error (RMSE). Since, in stereo panorama is the top-bottom fusion of left and right panorama, therefore we can assess the quality of stereo panorama by estimating the difference between stitched stereo panorama and unstitched left-right panoramas. For quantitative evaluation, we created three subsets of stereo panoramas generated by Lin et al. [66] system and our proposed system. Mathematically, these three image fidelity metrics can be written as follows:

(10) $$SSIM(x,y)=\frac{\left(2\mu_{x}\mu_{y}+c_{1}\right)\left(2\sigma_{xy}+c_{2}\right)}{\left(\mu_{x}^{2}+\mu_{y}^{2}+c_{1}\right)\left(\sigma_{x}^{2}+\sigma_{y}^{2}+c_{2}\right)}$$

Here, in Equation (10), the variable *μ_x_* represents the average of *x,* and *μ_y_* represents the average of *y,* where the variance of *x* is denoted by σ_x_*^2^*, and variance of y is denoted by σ_y_*^2^*. Similarly, σ*_xy_* represents the covariance of *x* and *y*, c1 and c2 are the two random variables that stabilized the division with weak denominator:

(11) $$MSE=\frac{1}{mn}\sum_{i=0}^{m-1}\sum_{j=0}^{n-1}{\left(I(i,j)-K(i,j)\right)}^{2}$$

Equation (11) is the mathematical representation of MSE, where *I(i,j)* is the reference stereo panoramic image, *K(i,j)* is the generated stereo panoramic image, m and n are the width and height of stereo panoramic image. The RMSE can be obtain by taking square root of MSE as given in Equation (12):

(12) $$RMSE=\sqrt{\frac{1}{mn}\sum_{i=0}^{m-1}\sum_{j=0}^{n-1}{\left(I(i,j)-K(i,j)\right)}^{2}}$$

(13) $$PSNR=10\cdot log_{10}\left(\frac{R^{2}}{MSE}\right)$$

**Figure 14:**
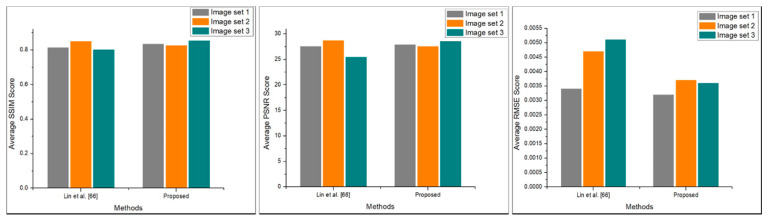
The obtained SSIM, PSNR, and RMSE score of our proposed method against Lin et al. [66] system.

Here in Equation (13), *R* is the maximum possible value of the stereo panoramic image. where the value of PSNR is obtained through dividing *R*^2^ by estimated MSE score. The obtained quantitative results are visualized in Figure 14, as it can be observe that the proposed system achieved better results in terms of RMSE and PSNR as compare to Lin et al. [66] system. Finally, we compare our proposed system with state-of-the-art stereo content generation systems in terms of number of cameras, panorama resolution, and stitching time. The conducted comparative study of our proposed system with existing systems are presented in Table 5. The comparison presented in Table 5 show that, the proposed system used the less number (only three cameras) of cameras as compare other stereo contents generation systems. Although, the resolution of generated stereo panorama is lower than first four comparative stereo contents generation systems, but in terms of hardware cost and the processing time the proposed system beaten rest of the stereo contents generation systems. Also, using a smaller number of cameras the proposed system can be used as a part of other system to generate high quality stereo contents thereby reducing time and computational complexity of the overall system.

## 5. Conclusions and Future Work

This paper presents an economical image acquisition system for a 360° mono-stereo panorama generation system. The proposed system deals with two different types of image acquisition modules, monoscopic and stereoscopic. For mono panorama generation, images are captured by six drone-mounted fisheye cameras that are placed in a circular rig with optimal overlapping gaps. For stereo panorama generation, we used only three cameras, two cameras are used to cover the front view and one camera is used to cover the rear view. The overlapping regions between adjacent cameras are sufficiently optimized for both image acquisition systems using the wider FOV of fisheye lens, and the resultant panoramic image has no unwanted artifacts. Furthermore, the proposed system is compared with existing mono and stereo contents generation system in terms of qualitative and quantitative perspectives. We also compare our proposed system in terms of hardware efficiency for both mono and stereo content generation. In future, we aim to extend our proposed system for video surveillance in smart cities, which will increase the spatial coverage range of the suspected area under observation using drone-mounted multi-camera intelligent sensors.

## Figures and Tables

**Table 1:** Descriptions of parameters, used for input and output operations in our proposed system.

Parameter	Description
Im	Mono image
Is	Stereo image
ICP	Initial camera parameters
CCP	Computer camera parameters
£_c_	Consistent features
H	Homography calculation function
Im_f_	Initial matched features
Fm_f_	Final matched features
RANSAC	Random sample consensus matching algorithm
ORB	Random sample consensus matching algorithm (Oriented FAST and rotated BRIEF) feature descriptor
Φ	Camera computation function
թ	Final panorama
w_i_	Wrapped image
Щ	Wrapping function
I_blend_	Blended image
βmulti-band	Image blending function
ζ_p_	Panorama straightening function

**Table 2:** Initial orientation of cameras for mono data acquisition.

Camera	Yaw	Pitch	Roll
Cam 1	0.0°	0.0°	0.0°
Cam 2	60.0°	0.0°	0.0°
Cam 3	120.0°	0.0°	0.0°
Cam 4	180.0°	0.0°	0.0°
Cam 5	−120.0°	0.0°	0.0°
Cam 6	−60.0°	0.0°	0.0°

**Table 3:** Field of views of stereo camera system.

Property	Field of View (FOV)
Front left camera	200°
Front right camera	200°
Rear camera	250°

**Table 4:** Comparison of the proposed system with state-of-the-art mono panorama generation systems.

System [Reference]	No of Cameras	Resolution	Stitching Artifacts	Stitching Time (s)
Lu et al. [64]	7	4 k	Extra black region	3.08
Mingxiu et al. [65]	6	4 k	Extra black region	2.98
Rodrigo et al. [63]	6	4 k	Parallax	3.01
Proposed system	6	4 k	Parallax-free	0.031

**Table 5:** Comparison of our proposed system with state-of-the-art stereo panorama generation hardware systems.

System [Reference]	No of Cameras	Panorama Resolution	Stitching Time (s)
Surround 360 [67]	17	8 k by 4 k	7.411
Google Jump [68]	16	8 k by4 k	6.532
Stereo cameras [69]	10	8 k by 4 k	4.072
NOKIA OZO [70]	8	8 k by 4 k	3.085
360 stereo cameras [71]	4	6 k by 3 k	2.984
Portable stereo cameras [66]	4	6 k by 3 k	2.413
Proposed system	3	6 k by 3 k	0.025

## References

[1] Thanh Le T., Jeong J., Ryu E.-S. (2019). Efficient Transcoding and Encryption for Live 360 CCTV System. Appl. Sci..

[2] Feriozzi R., Meschini A., Rossi D., Sicuranza F. (2019). VIRTUAL TOURS FOR SMART CITIES: A COMPARATIVE PHOTOGRAMMETRIC APPROACH FOR LOCATING HOT-SPOTS IN SPHERICAL PANORAMAS. Int. Arch. Photogramm. Remote. Sens. Spat. Inf. Sci..

[3] Shah A.A., Mustafa G., Ali Z., Anees T. (2019). Video Stitching with Localized 360o Model for Intelligent Car Parking Monitoring and Assistance System. IJCSNS.

[4] Demiralp K.O., Kurşun-Çakmak E.S., Bayrak S., Akbulut N., Atakan C., Orhan K. (2019). Trabecular structure designation using fractal analysis technique on panoramic radiographs of patients with bisphosphonate intake: A preliminary study. Oral Radiol..

[5] Wróżyński R., Pyszny K., Sojka M. (2020). Quantitative Landscape Assessment Using LiDAR and Rendered 360 Panoramic Images. Remote. Sens..

[6] Yong H., Huang J., Xiang W., Hua X., Zhang L. (2019). Panoramic background image generation for PTZ cameras. IEEE Trans. Image Process..

[7] Zia O., Kim J.H., Han K., Lee J.W. 360° Panorama Generation using Drone Mounted Fisheye Cameras. Proceedings of the 2019 IEEE International Conference on Consumer Electronics (ICCE).

[8] Krishnakumar K., Gandhi S.I. (2019). Video stitching using interacting multiple model based feature tracking. Multimedia Tools Appl..

[9] Qi J., Li G., Ju Z., Chen D., Jiang D., Tao B., Jiang G., Sun Y. Image stitching based on improved SURF algorithm. Proceedings of the International Conference on Intelligent Robotics and Applications.

[10] Sovetov K., Kim J.-S., Kim D. Online Panorama Image Generation for a Disaster Rescue Vehicle. Proceedings of the 2019 16th International Conference on Ubiquitous Robots (UR).

[11] Zhang J., Yin X., Luan J., Liu T. (2019). An improved vehicle panoramic image generation algorithm. Multimedia Tools Appl..

[12] Chen Z., Aksit D.C., Huang J., Jin H. (2019). Six-Degree of Freedom Video Playback of a Single Monoscopic 360-Degree Video. U.S. Patents.

[13] Bigioi P., Susanu G., Barcovschi I., Stec P., Murray L., Drimbarean A., Corcoran P. (2019). Stereoscopic (3d) Panorama Creation on Handheld Device. U.S. Patents.

[14] Zhang F., Nestares O. (2019). Generating Stereoscopic Light Field Panoramas Using Concentric Viewing Circles. U.S. Patents.

[15] Violante M.G., Vezzetti E., Piazzolla P. (2019). Interactive virtual technologies in engineering education: Why not 360° videos?. Int. J. Interact. Des. Manuf..

[16] Rupp M.A., Odette K.L., Kozachuk J., Michaelis J.R., Smither J.A., McConnell D.S. (2019). Investigating learning outcomes and subjective experiences in 360-degree videos. Comput. Educ..

[17] Kwon S. (2020). A CNN-Assisted Enhanced Audio Signal Processing for Speech Emotion Recognition. Sensors.

[18] Mustaqeem M., Sajjad M., Kwon S. (2020). Clustering Based Speech Emotion Recognition by Incorporating Learned Features and Deep BiLSTM. IEEE Access..

[19] Klippel A., Zhao J., Jackson K.L., La Femina P., Stubbs C., Wetzel R., Blair J., Wallgrün J.O., Oprean D. (2019). Transforming earth science education through immersive experiences: Delivering on a long held promise. J. Educ. Comput. Res..

[20] Mathew P.S., Pillai A.S. (2020). Role of Immersive (XR) Technologies in Improving Healthcare Competencies: A Review. Virtual and Augmented Reality in Education, Art, and Museums.

[21] Reyes M.E., Dillague S.G.O., Fuentes M.I.A., Malicsi C.A.R., Manalo D.C.F., Melgarejo J.M.T., Cayubit R.F.O. (2019). Self-Esteem and Optimism as Predictors of Resilience among Selected Filipino Active Duty Military Personnel in Military Camps. J. Posit. Psychol. Wellbeing.

[22] Wang K.-H., Lai S.-H. Object Detection in Curved Space for 360-Degree Camera. Proceedings of the ICASSP 2019–2019 IEEE International Conference on Acoustics, Speech and Signal Processing (ICASSP).

[23] Yang T., Li Z., Zhang F., Xie B., Li J., Liu L. (2019). Panoramic uav surveillance and recycling system based on structure-free camera array. IEEE Access..

[24] Heindl C., Pönitz T., Pichler A., Scharinger J. (2019). Large area 3D human pose detection via stereo reconstruction in panoramic cameras. arXiv.

[25] Qiu S., Zhou D., Du Y. (2019). The image stitching algorithm based on aggregated star groups. Signal. Image Video Process..

[26] Hu F., Li Y., Feng M. (2019). Continuous Point Cloud Stitch based on Image Feature Matching Constraint and Score. IEEE Trans. Intell. Vehicles.

[27] Bahraini M.S., Rad A.B., Bozorg M. (2019). SLAM in Dynamic Environments: A Deep Learning Approach for Moving Object Tracking Using ML-RANSAC Algorithm. Sensors.

[28] Shi H., Guo L., Tan S., Li G., Sun J. (2019). Improved parallax image stitching algorithm based on feature block. Symmetry.

[29] Chi L., Guan X., Shen X., Zhang H. Line-point feature based structure-preserving image stitching. Proceedings of the 2019 Chinese Automation Congress (CAC).

[30] Kekre H., Thepade S.D. Image blending in vista creation using Kekre’s LUV color space. Proceedings of the SPIT-IEEE Colloquium and International Conference.

[31] Gu F., Rzhanov Y. Optimal image blending for underwater mosaics. Proceedings of the OCEANS.

[32] Zhao W. (2016). Flexible image blending for image mosaicing with reduced artifacts. Int. J. Pattern Recognit. Artif. Intell..

[33] Shimizu T., Yoneyama A., Takishima Y. A fast video stitching method for motion-compensated frames in compressed video streams. Proceedings of the 2006 Digest of Technical Papers International Conference on Consumer Electronics.

[34] Kim H.-K., Lee K.-W., Jung J.-Y., Jung S.-W., Ko S.-J. (2011). A content-aware image stitching algorithm for mobile multimedia devices. IEEE Trans. Consum. Electron..

[35] Kim B.-S., Choi K.-A., Park W.-J., Kim S.-W., Ko S.-J. (2017). Content-preserving video stitching method for multi-camera systems. IEEE Trans. Consum. Electron..

[36] Guan L., Liu S., Chu J., Zhang R., Chen Y., Li S., Zhai L., Li Y., Xie H. (2019). A novel algorithm for estimating the relative rotation angle of solar azimuth through single-pixel rings from polar coordinate transformation for imaging polarization navigation sensors. Optik.

[37] Chen M., Tang Y., Zou X., Huang K., Li L., He Y. (2019). High-accuracy multi-camera reconstruction enhanced by adaptive point cloud correction algorithm. Opt. Lasers Eng..

[38] Tang Y., Li L., Wang C., Chen M., Feng W., Zou X., Huang K. (2019). Real-time detection of surface deformation and strain in recycled aggregate concrete-filled steel tubular columns via four-ocular vision. Robot. Comput. -Integr. Manuf..

[39] Lin G., Tang Y., Zou X., Li J., Xiong J. (2019). In-field citrus detection and localisation based on RGB-D image analysis. Biosyst. Eng..

[40] Tang Y., Lin Y., Huang X., Yao M., Huang Z., Zou X. (2020). Grand Challenges of Machine-Vision Technology in Civil Structural Health Monitoring. Artif. Intell. Evol..

[41] Joshi N., Kienzle W., Toelle M., Uyttendaele M., Cohen M.F. (2015). Real-time hyperlapse creation via optimal frame selection. Acm Trans. Graph. (TOG).

[42] Autostitch. http://matthewalunbrown.com/autostitch/autostitch.html.

[43] Panoweaver. https://www.easypano.com/panorama-software.html.

[44] Kolor Autopano. https://veer.tv/blog/kolor-autopano-create-a-panorama-with-autopano-progiga/.

[45] Tan L., Wang Y., Yu H., Zhu J. (2017). Automatic camera calibration using active displays of a virtual pattern. Sensors.

[46] Qu Z., Lin S.-P., Ju F.-R., Liu L. (2015). The improved algorithm of fast panorama stitching for image sequence and reducing the distortion errors. Math. Probl. Eng..

[47] Rublee E., Rabaud V., Konolige K., Bradski G. ORB: An efficient alternative to SIFT or SURF. Proceedings of the 2011 International conference on computer vision.

[48] Jeon H.-k., Jeong J.-m., Lee K.-y. An implementation of the real-time panoramic image stitching using ORB and PROSAC. Proceedings of the 2015 International SoC Design Conference (ISOCC).

[49] Wang M., Niu S., Yang X. A novel panoramic image stitching algorithm based on ORB. Proceedings of the 2017 International Conference on Applied System Innovation (ICASI).

[50] Brown M., Lowe D.G. (2017). Automatic panoramic image stitching using invariant features. Int. J. Comput. Vis..

[51] Din I., Anwar H., Syed I., Zafar H., Hasan L. (2014). Projector calibration for pattern projection systems. J. Appl. Res. Technol..

[52] Chaudhari K., Garg D., Kotecha K. (2017). An enhanced approach in Image Mosaicing using ORB Method with Alpha blending technique. Int. J. Adv. Res. Comput. Sci..

[53] Pandey A., Pati U.C. A novel technique for non-overlapping image mosaicing based on pyramid method. Proceedings of the 2013 Annual IEEE India Conference (INDICON).

[54] Dessein A., Smith W.A., Wilson R.C., Hancock E.R. Seamless texture stitching on a 3D mesh by Poisson blending in patches. Proceedings of the 2014 IEEE International Conference on Image Processing (ICIP).

[55] Allène C., Pons J.-P., Keriven R. Seamless image-based texture atlases using multi-band blending. Proceedings of the 2008 19th International Conference on Pattern Recognition.

[56] Burt P.J., Adelson E.H. (1983). A multiresolution spline with application to image mosaics. Acm Trans. Graph. (TOG).

[57] Li X., Zhu W., Zhu Q. Panoramic video stitching based on multi-band image blending. Proceedings of the Tenth International Conference on Graphics and Image Processing (ICGIP 2018).

[58] Kim H., Chae E., Jo G., Paik J. Fisheye lens-based surveillance camera for wide field-of-view monitoring. Proceedings of the 2015 IEEE International Conference on Consumer Electronics (ICCE).

[59] Saad M.A., Bovik A.C., Charrier C. DCT statistics model-based blind image quality assessment. Proceedings of the 2011 18th IEEE International Conference on Image Processing.

[60] Mittal A., Moorthy A.K., Bovik A.C. (2012). No-reference image quality assessment in the spatial domain. IEEE Trans. Image Process..

[61] Moorthy A.K., Bovik A.C. (2011). Blind image quality assessment: From natural scene statistics to perceptual quality. IEEE Trans. Image Process..

[62] Perazzi F., Sorkine-Hornung A., Zimmer H., Kaufmann P., Wang O., Watson S., Gross M. (2015). Panoramic video from unstructured camera arrays. Comput. Graph. Forum.

[63] Silva R.M., Feijó B., Gomes P.B., Frensh T., Monteiro D. Real time 360 video stitching and streaming. Proceedings of the ACM SIGGRAPH 2016 Posters.

[64] Lu Y., Wang K., Fan G. (2016). Photometric calibration and image stitching for a large field of view multi-camera system. Sensors.

[65] Lin M., Xu G., Ren X., Xu K. Cylindrical panoramic image stitching method based on multi-cameras. Proceedings of the 2015 IEEE International Conference on Cyber Technology in Automation, Control, and Intelligent Systems (CYBER).

[66] Lin H.-S., Chang C.-C., Chang H.-Y., Chuang Y.-Y., Lin T.-L., Ouhyoung M. (2018). A low-cost portable polycamera for stereoscopic 360 imaging. IEEE Trans. Circuits Syst. Video Technol..

[67] Facebook Surround 360. https://facebook360.fb.com/.

[68] Google Jump. https://arvr.google.com/.

[69] Amini A.S., Varshosaz M., Saadatseresht M. (2014). Evaluating a new stereo panorama system based on stereo cameras. Int. J. Sci. Res. Invent. New Ideas.

[70] Nokia Ozo. https://ozo.nokia.com/.

[71] Matzen K., Cohen M.F., Evans B., Kopf J., Szeliski R. (2017). Low-cost 360 stereo photography and video capture. Acm Trans. Graph. (TOG).

